# Supplementary material for: Genomic epidemiology and global diversity of the emerging bacterial pathogen Elizabethkingia anophelis
Source: Sci Rep. 2016 Jul 27;6:30379. doi: 10.1038/srep30379 (PMC4961963; doi:10.1038/srep30379)
Supplement: Supplementary Information [file srep30379-s1.doc]

Supplementary material

Genomic epidemiology and global diversity of the emerging bacterial pathogen *Elizabethkingia anophelis*

**Authors and affiliations**

Sebastien Breurec^1,2*^, Alexis Criscuolo^3^, Laure Diancourt^4^, Olaya Rendueles^5,6^, Mathias Vandenbogaert^4^, Virginie Passet^5,6^, Valérie Caro^4^, Eduardo P. C. Rocha^5,6^, Marie Touchon^5,6^, Sylvain Brisse^5,6*^

^1^Unité Environnement et Santé, Institut Pasteur de Guadeloupe, Pointe-à-Pitre, France, Faculté de Médecine Hyacinthe Bastaraud, Université des Antilles, Pointe-à-Pitre, France, Laboratoire de Microbiologie clinique et environnementale, Centre Hospitalier Universitaire de Pointe-à-Pitre/les Abymes, Pointe-à-Pitre, France

^2^Laboratoire de Microbiologie Clinique, Institut Pasteur de Bangui, Bangui, Central African Republic

^3^Institut Pasteur, Hub Bioinformatique et Biostatistique, C3BI, USR 3756 IP CNRS, Paris, France

^4^Unité Environnement et Risques Infectieux, Institut Pasteur, Paris, France

^5^Microbial Evolutionary Genomics, Institut Pasteur, Paris, France

^6^UMR 3525, CNRS, Paris, France

**TABLE S1. Genome characteristics of 20 strains belonging to *Elizabethkingia*** genus

| **Genomic identification/**  **Original identification** | **Strain (synonym)** | **Country/ Origin/ Year of isolation** | **Accession number** | **Total sequence length (bp)** | **Number of contigs** | **Contig N50** | **% GC** | **Gene** | **Reference** |
| --- | --- | --- | --- | --- | --- | --- | --- | --- | --- |
| *E. meningoseptica/ E. meningoseptica* | ATCC 13253T (NBRC 12535) | USA/ Human/ Unknown | ASAN00000000 | 3 796 928 | 115 | 64 581 | 36.6 | 3441 | doi:10.1128/genomeA.00444-13 |
| *E. miricola / E. miricola* | ATCC 33958 | Unknown/ Environment/ Unknown | JRFN00000000 | 4 578 109 | 75 | 158 315 | 35.9 | 3873 | doi: 10.1128/genomeA.00828-15. |
| *E. miricola / Elizabethkingia sp.* | BM10 | Unknown/ Unknown/ Unknown | CP011059 | 4 242 519 | 1 | 4 242 519 | 35.7 | 4293 | Unpublished |
| *E. anophelis/ E. meningoseptica* | 502 | Afghanistan/ Human/ Unknown | AVCQ00000000 | 3 960 658 | 21 | 482 386 | 35.5 | 3617 | doi:10.1128/genomeA.00355-14 |
| *E. anophelis/ E. meningoseptica* | B2D | Malaysia/ Human/ Unknown | JNCG00000000 | 3 936 249 | 50 | 128 773 | 35.5 | 3551 | Unpublished |
| *E. anophelis/ E. meningoseptica* | Endophthalmitis | Singapore/ Human/ Unknown | JSAA00000000 | 4 019 824 | 167 | 52 390 | 35.5 | 3729 | doi:10.1128/genomeA.01335-14 |
| *E. anophelis/ E. anophelis* | NUH11 | Singapore/ Environnement/ 2012 | ASYK00000000 | 4 091 484 | 59 | 264 278 | 35.6 | 3757 | doi: 10.1016/S0140-6736(13)61858-9 |
| *E. anophelis/ E. anophelis* | NUH6 | Singapore/ Environnement/ 2012 | ASYJ00000000 | 4 123 800 | 74 | 132 746 | 35.6 | 3812 | doi: 10.1016/S0140-6736(13)61858-9 |
| *E. anophelis/ E. anophelis* | NUHP2 | Singapore/ Human/ 2012 | ASYF00000000 | 4 334 646 | 59 | 422 755 | 35.5 | 3988 | doi: 10.1016/S0140-6736(13)61858-9 |
| *E. anophelis/ E. anophelis* | NUHP3 | Singapore/ Human/ 2012 | ASYG00000000 | 4 334 105 | 71 | 463 853 | 35.5 | 3985 | doi: 10.1016/S0140-6736(13)61858-9 |
| *E. anophelis/ E. anophelis* | NUHP1 | Singapore/ Human/ 2012 | CP007547 | 4 369 828 | 1 | 4 369 828 | 35.6 | 4016 | doi: 10.1016/S0140-6736(13)61858-9 |
| *E. anophelis/ E. anophelis* | NUH4 | Singapore/ Environnement/ 2012 | ASYI00000000 | 4 239 486 | 50 | 385 850 | 35.6 | 3912 | doi: 10.1016/S0140-6736(13)61858-9 |
| *E. anophelis/ E. anophelis* | NUH1 | Singapore/ Environnement/ 2012 | ASYH00000000 | 4 334 661 | 59 | 463 821 | 35.5 | 3993 | doi: 10.1016/S0140-6736(13)61858-9 |
| *E. anophelis/ E. anophelis* | Ag1 | Mosquito/ The Gambia/ Unknown | AHHG00000000 | 4 045 712 | 51 | 207 353 | 35.5 | 3722 | doi:10.1128/genomeA.01030-13 |
| *E. anophelis/ E. anophelis* | R26T | Mosquito /The Gambia/ Unknown | ANIW00000000 | 4 032 720 | 66 | 207 010 | 35.3 | 3726 | doi:10.1128/genomeA.01030-13 |
| *E. anophelis/ E. anophelis* | E18064 (V0378064) | Centrafrican Republic / Human/ 2011 | CCAB00000000 | 4 036 754 | 213 | 191 175 | 35.7 | 3648 | this study |
| *E. anophelis/ E. anophelis* | E27107 (Po0527107) | Centrafrican Republic / Human/ 2006 | CCAC00000000 | 4 032 057 | 89 | 161 028 | 35.5 | 3674 | this study |
| *E. anophelis/ E. anophelis* | HKU37 (PW2806) | Hong Kong/ Human/ 2012 | CBYD000000000 | 3 912 805 | 388 | 24 007 | 35.8 | 3588 | doi: 10.3201/eid2102.140623 |
| *E. anophelis/ E. anophelis* | HKU38 (PW2809) | Hong Kong/ Human/ 2012 | CBYE000000000 | 3 922 153 | 278 | 29 223 | 35.8 | 3598 | doi: 10.3201/eid2102.140623 |
| *E. anophelis/ E. anophelis* | HKU36 (PW2810) | Hong Kong/ Human/ 2012 | CBYF000000000 | 3 983 028 | 277 | 34 058 | 35.8 | 3601 | doi: 10.3201/eid2102.140623 |

**Table S2.** Average nucleotide identity values computed from 20 *Elizabethkingia* genome sequences.

| Strains | BM10 | E18064 | NUH4 | R26 | NUHP2 | NUH1 | PW2806 | NBRC12535 | NUH6 | NUHP1 | 502 | PW2809 | PW2810 | E27107 | NUH11 | B2D | NUHP3 | Endophthalmitis | Ag1 | ATCC33958 |
| --- | --- | --- | --- | --- | --- | --- | --- | --- | --- | --- | --- | --- | --- | --- | --- | --- | --- | --- | --- | --- |
| BM10 | --- | 91.1 | 91.1 | 91.1 | 91.1 | 91.1 | 91.1 | 80.0 | 91.2 | 91.1 | 91.1 | 91.1 | 91.0 | 91.1 | 91.1 | 91.1 | 91.1 | 91.0 | 91.1 | 96.3 |
| E18064 | 91.2 | --- | 97.9 | 97.6 | 97.9 | 97.9 | 97.9 | 79.9 | 97.8 | 97.9 | 97.7 | 97.9 | 97.2 | 100.0 | 97.7 | 97.3 | 97.9 | 97.1 | 97.6 | 91.2 |
| NUH4 | 90.7 | 97.4 | --- | 97.5 | 99.7 | 99.7 | 97.4 | 79.8 | 97.4 | 99.7 | 97.4 | 97.5 | 97.3 | 97.4 | 97.0 | 97.3 | 99.7 | 97.2 | 97.6 | 90.6 |
| R26 | 91.1 | 97.5 | 97.9 | --- | 97.9 | 97.9 | 98.1 | 79.9 | 97.7 | 97.9 | 97.6 | 98.2 | 97.3 | 97.5 | 97.5 | 97.4 | 97.9 | 97.4 | 100.0 | 91.2 |
| NUHP2 | 90.8 | 97.3 | 99.5 | 97.4 | --- | 100.0 | 97.4 | 79.9 | 97.6 | 100.0 | 97.4 | 97.4 | 97.3 | 97.3 | 97.2 | 97.4 | 100.0 | 97.2 | 97.4 | 90.9 |
| NUH1 | 90.7 | 97.3 | 99.5 | 97.4 | 100.0 | --- | 97.4 | 79.9 | 97.5 | 100.0 | 97.4 | 97.4 | 97.2 | 97.3 | 97.2 | 97.4 | 100.0 | 97.3 | 97.4 | 91.0 |
| PW2806 | 91.0 | 97.8 | 97.7 | 98.1 | 97.7 | 97.7 | --- | 79.9 | 97.5 | 97.7 | 97.5 | 99.7 | 97.2 | 97.8 | 97.6 | 97.3 | 97.7 | 97.2 | 98.1 | 91.1 |
| NBRC12535 | 79.9 | 79.8 | 79.9 | 79.9 | 79.9 | 79.9 | 79.9 | --- | 79.9 | 79.9 | 79.9 | 79.9 | 79.8 | 79.8 | 79.9 | 79.9 | 79.9 | 79.8 | 79.9 | 79.8 |
| NUH6 | 91.0 | 97.5 | 97.7 | 97.5 | 98.0 | 98.0 | 97.6 | 79.9 | --- | 98.0 | 97.5 | 97.6 | 97.2 | 97.5 | 99.7 | 97.2 | 98.0 | 97.1 | 97.5 | 91.0 |
| NUHP1 | 90.7 | 97.4 | 99.6 | 97.5 | 100.0 | 100.0 | 97.5 | 80.0 | 97.6 | --- | 97.4 | 97.5 | 97.3 | 97.4 | 97.3 | 97.4 | 100.0 | 97.2 | 97.6 | 91.0 |
| 502 | 91.1 | 97.7 | 97.6 | 97.5 | 97.6 | 97.6 | 97.5 | 79.9 | 97.5 | 97.6 | --- | 97.6 | 98.1 | 97.7 | 97.6 | 98.2 | 97.6 | 98.1 | 97.5 | 91.3 |
| PW2809 | 91.1 | 97.8 | 97.7 | 98.1 | 97.7 | 97.7 | 99.7 | 79.9 | 97.6 | 97.7 | 97.5 | --- | 97.2 | 97.8 | 97.6 | 97.3 | 97.7 | 97.2 | 98.1 | 91.2 |
| PW2810 | 91.0 | 97.3 | 97.4 | 97.3 | 97.3 | 97.3 | 97.2 | 79.9 | 97.2 | 97.3 | 98.1 | 97.3 | --- | 97.3 | 97.2 | 98.8 | 97.3 | 98.6 | 97.3 | 91.2 |
| E27107 | 91.2 | 100.0 | 97.9 | 97.7 | 97.9 | 97.9 | 97.9 | 79.9 | 97.9 | 97.9 | 97.7 | 97.9 | 97.2 | --- | 97.8 | 97.3 | 97.9 | 97.2 | 97.7 | 91.2 |
| NUH11 | 91.1 | 97.7 | 97.4 | 97.6 | 97.7 | 97.7 | 97.5 | 79.9 | 99.8 | 97.7 | 97.4 | 97.5 | 97.1 | 97.7 | --- | 97.2 | 97.7 | 97.0 | 97.6 | 91.0 |
| B2D | 91.0 | 97.3 | 97.4 | 97.4 | 97.4 | 97.4 | 97.3 | 79.9 | 97.2 | 97.4 | 98.2 | 97.3 | 98.9 | 97.3 | 97.3 | --- | 97.4 | 98.9 | 97.4 | 91.3 |
| NUHP3 | 90.7 | 97.3 | 99.5 | 97.4 | 100.0 | 100.0 | 97.4 | 79.8 | 97.6 | 100.0 | 97.4 | 97.4 | 97.2 | 97.3 | 97.3 | 97.4 | --- | 97.1 | 97.4 | 90.9 |
| Endophthalmitis | 90.9 | 97.1 | 97.2 | 97.2 | 97.2 | 97.2 | 97.1 | 79.8 | 97.0 | 97.2 | 98.0 | 97.1 | 98.6 | 97.1 | 97.1 | 98.9 | 97.2 | --- | 97.2 | 91.1 |
| Ag1 | 91.0 | 97.5 | 97.9 | 99.9 | 97.9 | 97.9 | 98.1 | 79.9 | 97.7 | 97.9 | 97.6 | 98.2 | 97.3 | 97.5 | 97.5 | 97.4 | 97.9 | 97.4 | --- | 91.1 |
| ATCC33958 | 96.1 | 90.9 | 90.6 | 91.1 | 90.9 | 90.9 | 90.7 | 79.7 | 90.7 | 90.9 | 91.0 | 90.7 | 91.1 | 91.0 | 90.7 | 91.1 | 90.9 | 91.0 | 91.0 | --- |

| **Table S3. Antimicrobial resistance-associated genomic features of *Elizabethkingia* genomes** | | | | | | | | | | | | |  |  |  |  |  |  |  |  |  |  |  |
| --- | --- | --- | --- | --- | --- | --- | --- | --- | --- | --- | --- | --- | --- | --- | --- | --- | --- | --- | --- | --- | --- | --- | --- |
|  |  |  |  |  |  |  |  |  |  |  |  | ***E. anophelis*** | |  |  |  |  |  |  |  | ***E. miricola*** |  | ***E. meningoseptica*** |
| **Description** | **Resfam Family Name** | **Resfam ID** | E27107 | E18064 | NUH6 | NUH11 | R26 | Ag1 | PW2809 | PW2806 | NUH1 | NUH4 | NUHP1 | NUHP3 | NUHP2 | PW2810 | B2D | 502 |  | BM10 | ATCC33958 |  | NBRC1235 |
| tetracycline resistance MFS efflux pump: selectively pump out tetracycline or tetracycline derivatives | Tetracycline_Resistance_MFS_Efflux_Pump | RF0104-RF0134 | 1 | 1 | 1 | 1 | 1 | 1 | 1 | 1 | 1 | 1 | 1 | 1 | 1 | 1 | 1 | 1 |  | 1 | 1 |  | 1 |
| Subclass B1 (metallo-) beta-lactamase hydrolize penicillins, cephalosporins and carbapenems | SubclassB1 | RF0123 | 1 | 1 | 1 | 1 | 1 | 1 | 1 | 1 | 1 | 1 | 1 | 1 | 1 | 1 | 1 | 1 |  | 1 | 1 |  | 1 |
| resistance-nodulation-cell division (RND) antibiotic efflux pump | RNDAntibioticEffluxPump | RF0115 | 1 | 1 | 1 | 1 | 1 | 1 | 1 | 1 | 1 | 1 | 1 | 1 | 1 | 1 | 1 | 1 |  | 1 | 1 |  | 1 |
| ATP-binding cassette (ABC) antibiotic efflux pump | ABCAntibioticEffluxPump | RF0007 | 1 | 1 | 1 | 1 | 1 | 1 | 1 | 1 | 1 | 1 | 1 | 1 | 1 | 1 | 1 | 1 |  | 1 | 1 |  | 1 |
| ATP-binding cassette (ABC) antibiotic efflux pump | ABCAntibioticEffluxPump | RF0007 | 1 | 1 | 1 | 1 | 1 | 1 | 1 | 1 | 1 | 1 | 1 | 1 | 1 | 1 | 1 | 1 |  | 1 | 1 |  | 1 |
| Subclass B3 (metallo-) beta-lactamase hydrolize penicillins, cephalosporins and carbapenems / GOB beta-lactamase (subclass B3 (metallo-) beta-lactamase) | SubclassB3 - GOB | RF0078-RF0125 | 1 | 1 | 1 | 1 | 1 | 1 | 1 | 1 | 1 | 1 | 1 | 1 | 1 | 1 | 1 | 1 |  | 1 | 1 |  | 1 |
| mexH: membrane fusion protein of the efflux complex MexGHI-OpmD | MexH | RF0099 | 1 | 1 | 1 | 1 | 1 | 1 | 1 | 1 | 1 | 1 | 1 | 1 | 1 | 1 | 1 | 1 |  | 1 | 1 |  | 1 |
| Beta-lactamase B (BlaB) (subclass B1 (metallo-) beta-lactamase) | BlaB | RF0041 | 1 | 1 | 1 | 1 | 1 | 1 | 1 | 0 | 1 | 1 | 1 | 1 | 1 | 0 | 1 | 1 |  | 1 | 1 |  | 1 |
| chloramphenicol acetyltransferase (CAT) | Chloramphenicol_Acetyltransferase_CAT | RF0050 | **2** | **2** | 1 | 1 | 1 | 1 | 1 | 1 | 1 | 1 | 1 | 1 | 1 | 1 | 1 | 1 |  | 1 | 1 |  | 1 |
| ATP-binding cassette (ABC) antibiotic efflux pump | ABCAntibioticEffluxPump | RF0007 | 1 | 1 | 1 | 1 | 1 | 1 | 1 | 1 | 1 | 1 | 1 | 1 | 1 | 1 | 1 | 1 |  | 1 | 1 |  | 1 |
| PF13523.1 Acetyltransferase (GNAT) domain | Acetyltransf_8 | RF0115 | 1 | 1 | 1 | 1 | 1 | 1 | 1 | 1 | 1 | 1 | 1 | 1 | 1 | 1 | 1 | 1 |  | 1 | 1 |  | 1 |
| mexE: membrane fusion protein of the MexEF-OprN multidrug efflux complex | MexE | RF0098 | 1 | 1 | 1 | 1 | 1 | 1 | 1 | 1 | 1 | 1 | 1 | 1 | 1 | 1 | 1 | 1 |  | 1 | 1 |  | 1 |
| resistance-nodulation-cell division (RND) antibiotic efflux pump | RNDAntibioticEffluxPump | RF0115 | 1 | 1 | 0 | 0 | 1 | 1 | 1 | 1 | 1 | 1 | 1 | 1 | 1 | 1 | 1 | 1 |  | 1 | 1 |  | 1 |
| Class A beta-lactamase | ClassA | RF0053 | 1 | 1 | 1 | 1 | 1 | 1 | 0 | 1 | 1 | 1 | 1 | 1 | 1 | 1 | 1 | 1 |  | 1 | 1 |  | 1 |
| resistance-nodulation-cell division (RND) antibiotic efflux pump | RNDAntibioticEffluxPump | RF0115 | 1 | 1 | 1 | 1 | 1 | 1 | 1 | 1 | 1 | 1 | 1 | 1 | 1 | 1 | 1 | 1 |  | 1 | 1 |  | 1 |
| Class A beta-lactamase | ClassA | RF0053 | 1 | 1 | 1 | 1 | 1 | 1 | 1 | 1 | 1 | 1 | 1 | 1 | 1 | 1 | 1 | 1 |  | 0 | 1 |  | 1 |
| resistance-nodulation-cell division (RND) antibiotic efflux pump | RNDAntibioticEffluxPump | RF0115 | 1 | 1 | 1 | 1 | 1 | 1 | 1 | 1 | 1 | 1 | 1 | 1 | 1 | 1 | 1 | 1 |  | 1 | 1 |  | 1 |
| mexH: membrane fusion protein of the efflux complex MexGHI-OpmD | MexH | RF0099 | 1 | 1 | 1 | 1 | 1 | 1 | 1 | 1 | 1 | 1 | 1 | 1 | 1 | 0 | 0 | 0 |  | 0 | 0 |  | 0 |
| resistance-nodulation-cell division (RND) antibiotic efflux pump | RNDAntibioticEffluxPump | RF0115 | 1 | 1 | 1 | 1 | 1 | 1 | 1 | 1 | 1 | 1 | 1 | 1 | 1 | 0 | 0 | 0 |  | 0 | 0 |  | 0 |
| ATP-binding cassette (ABC) antibiotic efflux pump | ABCAntibioticEffluxPump | RF0007 | 0 | 0 | 0 | 0 | 0 | 1 | 0 | 0 | 0 | 0 | 0 | 0 | 0 | 1 | 1 | 0 |  | 0 | 1 |  | 0 |
| tetX: tetracycline inactivation enzyme | TetX | RF0136 | **1** | **1** | 0 | 0 | 0 | 0 | 0 | 0 | 0 | 1 | 0 | 0 | 0 | 0 | 0 | 0 |  | 0 | 0 |  | 0 |
| Aminoglycoside Acetyltransferase (AAC3-I) | AAC3-I | RF0003 | **1** | **1** | 1 | 0 | 0 | 0 | 0 | 0 | 0 | 0 | 0 | 0 | 0 | 0 | 0 | 0 |  | 0 | 0 |  | 0 |
| A grouping of related NDM and CcrA beta-lactamases / Subclass B1 (metallo-) beta-lactamase hydrolize penicillins, cephalosporins and carbapenems | SubclassB1- NDM-CcrA | RF0108-RF0123 | **2** | **2** | 0 | 0 | 0 | 0 | 0 | 0 | 0 | 0 | 0 | 0 | 0 | 0 | 0 | 0 |  | 0 | 0 |  | 0 |
| **TOTAL** |  |  | 24 | 24 | 19 | 18 | 19 | 20 | 18 | 18 | 19 | 20 | 19 | 19 | 19 | 17 | 18 | 17 |  | 16 | 18 |  | 17 |

|  |  |  |  |
| --- | --- | --- | --- |

| **Table S4. Predicted virulence factors in *Elizabethkingia* genomes** | | | | | | | | |  |  |  |  |  |  |  |  |  |  |  |  |  |  |  |  |
| --- | --- | --- | --- | --- | --- | --- | --- | --- | --- | --- | --- | --- | --- | --- | --- | --- | --- | --- | --- | --- | --- | --- | --- | --- |
|  |  |  |  |  |  | ***E. anophelis*** | | | |  |  |  |  |  |  |  |  |  |  |  |  | ***E. miricola*** | | ***E. meningoseptica*** |
|  |  |  |  |  |  | NUH6 | NUH11 | E27107 | E18064 | R26 | Ag1 | PW2809 | PW2806 | NUH1 | NUH4 | NUHP1 | NUHP3 | NUHP2 | PW2810 | B2D | 502 | BM10 | ATCC33958 | NBRC1235 |
| Family | VFDB-ID# | VFDB-name | Annotation-VFDB-gene | Anntotation-Gene-Genome | Total No. of identified proteins | ELAN006 | ELAN004 | ELAN013 | ELAN015 | ELAN014 | ELAN002 | ELAN010 | ELAN011 | ELAN003 | ELAN005 | ELAN007 | ELAN009 | ELAN008 | ELAN012 | ELAN016 | ELAN001 | ELMI001 | ELMI002 | ELME001 |
| Fam13 | VFG011430(gi:17987758) | (acpXL) | (acpXL) acyl carrier protein [LPS (CVF383)] [Brucella melitensis bv. 1 str. 16M] | acyl carrier protein | 19 | 1 | 1 | 1 | 1 | 1 | 1 | 1 | 1 | 1 | 1 | 1 | 1 | 1 | 1 | 1 | 1 | 1 | 1 | 1 |
| Fam30 | VFG000696(gi:30962644) | (bexA) | (bexA) ATP-dependent polysaccharide export protein BexA [Capsule (VF0043)] [Haemophilus influenzae str. 1007] | ABC transporter | 19 | 1 | 1 | 1 | 1 | 1 | 1 | 1 | 1 | 1 | 1 | 1 | 1 | 1 | 1 | 1 | 1 | 1 | 1 | 1 |
| Fam82 | VFG000037(gi:33591350) | (bplB) | (bplB) probable acetyltransferase [LPS (VF0033)] [Bordetella pertussis Tohama I] | WxcM-like protein | 14 | 1 | 1 | 1 | 1 | 1 | 1 | 1 | 1 | 1 | 1 | 1 | 1 | 1 | 0 | 0 | 0 | 1 | 0 | 0 |
| Fam77 | VFG000036(gi:33591349) | (bplC) | (bplC) lipopolysaccharide biosynthesis protein [LPS (VF0033)] [Bordetella pertussis Tohama I] | UDP-2-acetamido-2-deoxy-3-oxo-D-glucuronate aminotransferase | 19 | 1 | 1 | 1 | 1 | 1 | 1 | 1 | 1 | 1 | 1 | 1 | 1 | 1 | 1 | 1 | 1 | 1 | 1 | 1 |
| Fam85 | VFG000032(gi:33591345) | (bplG) | (bplG) probable sugar transferase [LPS (VF0033)] [Bordetella pertussis Tohama I] | glycosyl transferase | 6 | 0 | 0 | 0 | 0 | 0 | 0 | 0 | 0 | 1 | 1 | 1 | 1 | 1 | 0 | 0 | 0 | 0 | 1 | 0 |
| Fam108 | VFG002440(gi:53722543) | (bprB) | (bprB) two-component response regulator [Bsa T3SS (VF0428)] [Burkholderia pseudomallei K96243] | LuxR family transcriptional regulator | 3 | 0 | 0 | 0 | 0 | 0 | 0 | 0 | 0 | 0 | 0 | 0 | 0 | 0 | 0 | 0 | 0 | 0 | 0 | 3 |
| Fam53 | VFG001931(gi:15792793) | (cadF) | (cadF) outer membrane fibronectin-binding protein [CadF (VF0322)] [Campylobacter jejuni subsp. jejuni NCTC 11168] | flagellar motor protein MotB | 18 | 1 | 1 | 1 | 1 | 1 | 1 | 1 | 1 | 1 | 1 | 1 | 1 | 1 | 1 | 1 | 1 | 1 | 1 | 0 |
| Fam20 | VFG001300(gi:21281856) | (cap8D) | (cap8D) capsular polysaccharide synthesis enzyme Cap8D [Capsule (VF0003)] [Staphylococcus aureus subsp. aureus MW2] | capsule biosynthesis protein CapD | 18 | 1 | 1 | 1 | 1 | 1 | 1 | 1 | 1 | 1 | 1 | 1 | 1 | 1 | 1 | 1 | 1 | 1 | 0 | 1 |
| Fam92 | VFG001301(gi:21281857) | (cap8E) | (cap8E) capsular polysaccharide synthesis enzyme Cap8E [Capsule (VF0003)] [Staphylococcus aureus subsp. aureus MW2] | UDP-glucose 4-epimerase | 2 | 1 | 1 | 0 | 0 | 0 | 0 | 0 | 0 | 0 | 0 | 0 | 0 | 0 | 0 | 0 | 0 | 0 | 0 | 0 |
| Fam26 | VFG001301(gi:21281857) | (cap8E) | (cap8E) capsular polysaccharide synthesis enzyme Cap8E [Capsule (VF0003)] [Staphylococcus aureus subsp. aureus MW2] | putative polysaccharide biosynthesis protein CapE-like | 3 | 0 | 0 | 1 | 1 | 0 | 0 | 0 | 0 | 0 | 0 | 0 | 0 | 0 | 0 | 0 | 1 | 0 | 0 | 0 |
| Fam27 | VFG001303(gi:21281859) | (cap8G) | (cap8G) capsular polysaccharide synthesis enzyme Cap8G [Capsule (VF0003)] [Staphylococcus aureus subsp. aureus MW2] | Capsular polysaccharide synthesis enzyme Cap5G | 3 | 0 | 0 | 1 | 1 | 0 | 0 | 0 | 0 | 0 | 0 | 0 | 0 | 0 | 0 | 0 | 1 | 0 | 0 | 0 |
| Fam22 | VFG001306(gi:21281862 | (cap8J) | (cap8J) capsular polysaccharide synthesis enzyme Cap8J [Capsule (VF0003)] [Staphylococcus aureus subsp. aureus MW2] | hypothetical protein C874_10240 | 1 | 0 | 0 | 0 | 0 | 0 | 0 | 0 | 0 | 0 | 0 | 0 | 0 | 0 | 0 | 0 | 1 | 0 | 0 | 0 |
| Fam45 | VFG001306(gi:21281862) | (cap8J) | (cap8J) capsular polysaccharide synthesis enzyme Cap8J [Capsule (VF0003)] [Staphylococcus aureus subsp. aureus MW2] | chloramphenicol acetyltransferase | 21 | 1 | 1 | 2 | 2 | 1 | 1 | 1 | 1 | 1 | 1 | 1 | 1 | 1 | 1 | 1 | 1 | 1 | 1 | 1 |
| Fam125 | VFG001306(gi:21281862) | (cap8J) | (cap8J) capsular polysaccharide synthesis enzyme Cap8J [Capsule (VF0003)] [Staphylococcus aureus subsp. aureus MW2] | hypothetical protein | 1 | 0 | 0 | 0 | 0 | 0 | 0 | 0 | 0 | 0 | 0 | 0 | 0 | 0 | 0 | 0 | 0 | 0 | 1 | 0 |
| Fam3 | VFG044172(gi:26250140) | (chuV) | (chuV) ATP-binding hydrophilic protein ChuV [Chu (VF0227)] [Escherichia coli CFT073 | heme ABC transporter ATP-binding protein | 19 | 1 | 1 | 1 | 1 | 1 | 1 | 1 | 1 | 1 | 1 | 1 | 1 | 1 | 1 | 1 | 1 | 1 | 1 | 1 |
| Fam83 | VFG001936(gi:15792460) | (Cj1135) | (Cj1135) glucosyltransferase [LOS (VF0326)] [Campylobacter jejuni subsp. jejuni NCTC 11168] | family 2 glycosyl transferase | 4 | 0 | 0 | 0 | 0 | 1 | 1 | 1 | 1 | 0 | 0 | 0 | 0 | 0 | 0 | 0 | 0 | 0 | 0 | 0 |
| Fam90 | VFG001937(gi:15792461) | (Cj1136) | (Cj1136) glucosyltransferase [LOS (VF0326)] [Campylobacter jejuni subsp. jejuni NCTC 11168] | hypothetical protein | 5 | 0 | 0 | 0 | 0 | 0 | 0 | 0 | 0 | 1 | 1 | 0 | 1 | 1 | 0 | 1 | 0 | 0 | 0 | 0 |
| Fam91 | VFG001937(gi:15792461) | (Cj1136) | (Cj1136) glucosyltransferase [LOS (VF0326)] [Campylobacter jejuni subsp. jejuni NCTC 11168] | hypothetical protein | 2 | 1 | 1 | 0 | 0 | 0 | 0 | 0 | 0 | 0 | 0 | 0 | 0 | 0 | 0 | 0 | 0 | 0 | 0 | 0 |
| Fam110 | VFG001937(gi:15792461) | (Cj1136) | (Cj1136) glucosyltransferase [LOS (VF0326)] [Campylobacter jejuni subsp. jejuni NCTC 11168] | hypothetical protein | 1 | 0 | 0 | 0 | 0 | 0 | 0 | 0 | 0 | 0 | 0 | 0 | 0 | 0 | 0 | 0 | 0 | 0 | 0 | 1 |
| Fam95 | VFG001939(gi:15792463) | (Cj1138) | (Cj1138) glycosyltransferase [LOS (VF0326)] [Campylobacter jejuni subsp. jejuni NCTC 11168] | Glycosyltransferase | 1 | 0 | 0 | 0 | 0 | 0 | 0 | 0 | 0 | 0 | 0 | 1 | 0 | 0 | 0 | 0 | 0 | 0 | 0 | 0 |
| Fam24 | VFG001989(gi:15792734) | (Cj1416c) | (Cj1416c) sugar nucleotidyltransferase [Capsule (VF0323)] [Campylobacter jejuni subsp. jejuni NCTC 11168] | nucleotidyl transferase | 1 | 0 | 0 | 0 | 0 | 0 | 0 | 0 | 0 | 0 | 0 | 0 | 0 | 0 | 0 | 0 | 1 | 0 | 0 | 0 |
| Fam120 | VFG001968(gi:15792758) | (Cj1440c) | (Cj1440c) sugar transferase [Capsule (VF0323)] [Campylobacter jejuni subsp. jejuni NCTC 11168] | Putative glycosyltransferase EpsE | 1 | 0 | 0 | 0 | 0 | 0 | 0 | 0 | 0 | 0 | 0 | 0 | 0 | 0 | 0 | 0 | 0 | 1 | 0 | 0 |
| Fam49 | VFG000079(gi:16802278) | (clpC) | (clpC) endopeptidase Clp ATP-binding chain C [ClpC (VF0072)] [Listeria monocytogenes EGD-e] | ATP-dependent Clp protease ClpC | 19 | 1 | 1 | 1 | 1 | 1 | 1 | 1 | 1 | 1 | 1 | 1 | 1 | 1 | 1 | 1 | 1 | 1 | 1 | 1 |
| Fam63 | VFG000079(gi:16802278) | (clpC) | (clpC) endopeptidase Clp ATP-binding chain C [ClpC (VF0072)] [Listeria monocytogenes EGD-e] | ATP-dependent protease, Hsp 100, part of multi-chaperone system with DnaK, DnaJ, and GrpE | 19 | 1 | 1 | 1 | 1 | 1 | 1 | 1 | 1 | 1 | 1 | 1 | 1 | 1 | 1 | 1 | 1 | 1 | 1 | 1 |
| Fam66 | VFG000077(gi:16804506) | (clpP) | (clpP) ATP-dependent Clp protease proteolytic subunit [ClpP (VF0074)] [Listeria monocytogenes EGD-e] | ATP-dependent Clp protease proteolytic subunit (Endopeptidase Clp) (Caseinolytic protease) | 19 | 1 | 1 | 1 | 1 | 1 | 1 | 1 | 1 | 1 | 1 | 1 | 1 | 1 | 1 | 1 | 1 | 1 | 1 | 1 |
| Fam71 | VFG002276(gi:18309155) | (colA) | (colA) collagenase [kappa-toxin (VF0388)] [Clostridium perfringens str. 13] | PKD domain-containing protein | 19 | 1 | 1 | 1 | 1 | 1 | 1 | 1 | 1 | 1 | 1 | 1 | 1 | 1 | 1 | 1 | 1 | 1 | 1 | 1 |
| Fam81 | VFG001369(gi:15900279) | (cps4E) | (cps4E) capsular polysaccharide biosynthesis protein Cps4E [Capsule (VF0144)] [Streptococcus pneumoniae TIGR4] | sugar transferase | 5 | 0 | 0 | 0 | 0 | 1 | 1 | 0 | 0 | 0 | 0 | 0 | 0 | 0 | 1 | 1 | 0 | 0 | 0 | 1 |
| Fam52 | VFG002190(gi:29376987) | (cpsA) | (cpsA) undecaprenyl diphosphate synthase [Capsule (VF0361)] [Enterococcus faecalis V583] | undecaprenyl pyrophosphate synthetase (di-trans,poly-cis-decaprenylcistransferase) | 19 | 1 | 1 | 1 | 1 | 1 | 1 | 1 | 1 | 1 | 1 | 1 | 1 | 1 | 1 | 1 | 1 | 1 | 1 | 1 |
| Fam50 | VFG002189(gi:29376986) | (cpsB) | (cpsB) phosphatidate cytidylyltransferase [Capsule (VF0361)] [Enterococcus faecalis V583] | phosphatidate cytidylyltransferase | 19 | 1 | 1 | 1 | 1 | 1 | 1 | 1 | 1 | 1 | 1 | 1 | 1 | 1 | 1 | 1 | 1 | 1 | 1 | 1 |
| Fam21 | VFG001349(gi:22537330) | (cpsD) | (cpsD) CpsD autokinase [Capsule (VF0274)] [Streptococcus agalactiae 2603V/R] | capsular biosynthesis protein | 19 | 1 | 1 | 1 | 1 | 1 | 1 | 1 | 1 | 1 | 1 | 1 | 1 | 1 | 1 | 1 | 1 | 1 | 1 | 1 |
| Fam88 | VFG001341(gi:22537322) | (cpsJ) | (cpsJ) glycosyl transferase CpsJ(V) [Capsule (VF0274)] [Streptococcus agalactiae 2603V/R] | putative glycosyltransferase protein | 5 | 0 | 0 | 0 | 0 | 0 | 0 | 0 | 0 | 1 | 1 | 1 | 1 | 1 | 0 | 0 | 0 | 0 | 0 | 0 |
| Fam89 | VFG002181(gi:29376978) | (cpsJ) | (cpsJ) ABC transporter, ATP-binding protein [Capsule (VF0361)] [Enterococcus faecalis V583] | Polysaccharide ABC transporter, ATP-binding protei | 6 | 0 | 0 | 0 | 0 | 0 | 0 | 0 | 0 | 1 | 1 | 1 | 1 | 1 | 0 | 1 | 0 | 0 | 0 | 0 |
| Fam101 | VFG001341(gi:22537322) | (cpsJ) | (cpsJ) glycosyl transferase CpsJ(V) [Capsule (VF0274)] [Streptococcus agalactiae 2603V/R] | hypothetical protein | 1 | 0 | 0 | 0 | 0 | 0 | 0 | 0 | 0 | 0 | 0 | 0 | 0 | 0 | 0 | 1 | 0 | 0 | 0 | 0 |
| Fam111 | VFG001341(gi:22537322) | (cpsJ) | (cpsJ) glycosyl transferase CpsJ(V) [Capsule (VF0274)] [Streptococcus agalactiae 2603V/R] | hypothetical protein | 1 | 0 | 0 | 0 | 0 | 0 | 0 | 0 | 0 | 0 | 0 | 0 | 0 | 0 | 0 | 0 | 0 | 0 | 0 | 1 |
| Fam126 | VFG002181(gi:29376978) | (cpsJ) | (cpsJ) ABC transporter, ATP-binding protein [Capsule (VF0361)] [Enterococcus faecalis V583] | hypothetical protein | 1 | 0 | 0 | 0 | 0 | 0 | 0 | 0 | 0 | 0 | 0 | 0 | 0 | 0 | 0 | 0 | 0 | 0 | 1 | 0 |
| Fam35 | VFG001342(gi:22537323) | (cpsO) | (cpsO) glycosyl transferase CpsO(V) [Capsule (VF0274)] [Streptococcus agalactiae 2603V/R] | glycosyl transferase family 2 | 19 | 1 | 1 | 1 | 1 | 1 | 1 | 1 | 1 | 1 | 1 | 1 | 1 | 1 | 1 | 1 | 1 | 1 | 1 | 1 |
| Fam68 | VFG001342(gi:22537323) | (cpsO) | (cpsO) glycosyl transferase CpsO(V) [Capsule (VF0274)] [Streptococcus agalactiae 2603V/R] | glycosyl transferase family 2 | 19 | 1 | 1 | 1 | 1 | 1 | 1 | 1 | 1 | 1 | 1 | 1 | 1 | 1 | 1 | 1 | 1 | 1 | 1 | 1 |
| Fam119 | VFG001342(gi:22537323) | (cpsO) | (cpsO) glycosyl transferase CpsO(V) [Capsule (VF0274)] [Streptococcus agalactiae 2603V/R] | putative glycosyltransferase EpsJ | 1 | 0 | 0 | 0 | 0 | 0 | 0 | 0 | 0 | 0 | 0 | 0 | 0 | 0 | 0 | 0 | 0 | 1 | 0 | 0 |
| Fam105 | VFG000457(gi:16764499) | (csgB) | (csgB) minor curlin subunit precursor, curli nucleator protein CsgB [Agf (VF0103)] [Salmonella enterica subsp. enterica serovar Typhimurium str. LT2] | hypothetical protein | 1 | 0 | 0 | 0 | 0 | 0 | 0 | 0 | 0 | 0 | 0 | 0 | 0 | 0 | 0 | 0 | 0 | 0 | 0 | 1 |
| Fam55 | VFG004125(gi:16764498) | (csgD) | (csgD) DNA-binding transcriptional regulator CsgD [curli fibers/thin aggregative fimbriae (AGF) (AI094)] [Salmonella enterica subsp. enterica serovar Typhimurium str. LT2] | LuxR family transcriptional regulator | 19 | 1 | 1 | 1 | 1 | 1 | 1 | 1 | 1 | 1 | 1 | 1 | 1 | 1 | 1 | 1 | 1 | 1 | 1 | 1 |
| Fam106 | VFG000462(gi:16764495) | (csgG) | (csgG) curli production assembly/transport protein CsgG [Agf (VF0103)] [Salmonella enterica subsp. enterica serovar Typhimurium str. LT2] | Curli production assembly/transport component CsgG | 1 | 0 | 0 | 0 | 0 | 0 | 0 | 0 | 0 | 0 | 0 | 0 | 0 | 0 | 0 | 0 | 0 | 0 | 0 | 1 |
| Fam47 | VFG005767(gi:22536831) | (cylG) | (cylG) 3-ketoacyl-ACP-reductase CylG [Beta-hemolysin/cytolysin (CVF171)] [Streptococcus agalactiae 2603V/R] | 3-oxoacyl-(acyl-carrier-protein) reductase | 19 | 1 | 1 | 1 | 1 | 1 | 1 | 1 | 1 | 1 | 1 | 1 | 1 | 1 | 1 | 1 | 1 | 1 | 1 | 1 |
| Fam70 | VFG002064(gi:15595276) | (dotU1) | (dotU1) type VI secretion system protein DotU [HSI-I (VF0334)] [Pseudomonas aeruginosa PAO1] | OmpA/MotB domain protein | 19 | 1 | 1 | 1 | 1 | 1 | 1 | 1 | 1 | 1 | 1 | 1 | 1 | 1 | 1 | 1 | 1 | 1 | 1 | 1 |
| Fam127 | VFG002364(gi:123443282) | (fcl) | (fcl) GDP-fucose synthetase [O-antigen (VF0392)] [Yersinia enterocolitica subsp. enterocolitica 8081] | GDP-L-fucose synthase | 1 | 0 | 0 | 0 | 0 | 0 | 0 | 0 | 0 | 0 | 0 | 0 | 0 | 0 | 0 | 0 | 0 | 0 | 1 | 0 |
| Fam86 | VFG001956(gi:15792746) | (fcl) | (fcl) GDP-L-fucose synthetase [Capsule (VF0323)] [Campylobacter jejuni subsp. jejuni NCTC 11168] | GDP-4-keto-6-deoxy-D-mannose-3, 5-epimerase-4-reductase | 6 | 0 | 0 | 0 | 0 | 0 | 0 | 0 | 0 | 1 | 1 | 1 | 1 | 1 | 0 | 0 | 0 | 0 | 1 | 0 |
| Fam36 | VFG000923(gi:26246561) | (fepA) | (fepA) ferrienterobactin outer membrane transporter [Enterobactin (VF0228)] [Escherichia coli CFT073] | TonB-denpendent receptor | 17 | 1 | 1 | 1 | 1 | 1 | 1 | 1 | 1 | 1 | 1 | 1 | 1 | 1 | 1 | 1 | 1 | 0 | 1 | 0 |
| Fam46 | VFG043319(gi:52841089) | (fleQ) | (fleQ) transcriptional regulator FleQ [polar flagella (AI149)] [Legionella pneumophila subsp. pneumophila str. Philadelphia 1] | Transcriptional regulator | 19 | 1 | 1 | 1 | 1 | 1 | 1 | 1 | 1 | 1 | 1 | 1 | 1 | 1 | 1 | 1 | 1 | 1 | 1 | 1 |
| Fam1 | VFG001249(gi:15596296) | (fleR) | (fleR) two-component response regulator [Flagella (VF0273)] [Pseudomonas aeruginosa PAO1] | LytTR family transcriptional regulator | 17 | 1 | 1 | 1 | 1 | 1 | 1 | 1 | 1 | 1 | 1 | 1 | 1 | 1 | 1 | 1 | 1 | 0 | 1 | 0 |
| Fam65 | VFG001249(gi:15596296) | (fleR) | (fleR) two-component response regulator [Flagella (VF0273)] [Pseudomonas aeruginosa PAO1] | chemotaxis protein CheY | 19 | 1 | 1 | 1 | 1 | 1 | 1 | 1 | 1 | 1 | 1 | 1 | 1 | 1 | 1 | 1 | 1 | 1 | 1 | 1 |
| Fam79 | VFG011946(gi:15792351) | (flgR) | (flgR) sigma-54 associated transcriptional activator [Pse5Ac7Ac, Pse5Ac7Am, Pse8OAc, Pse5Am7AcGlnAc (AI151)] [Campylobacter jejuni subsp. | transcriptional regulator | 11 | 0 | 0 | 0 | 0 | 1 | 1 | 1 | 1 | 1 | 1 | 1 | 1 | 1 | 0 | 0 | 0 | 1 | 1 | 0 |
| Fam103 | VFG011946(gi:15792351) | (flgR) | (flgR) sigma-54 associated transcriptional activator [Pse5Ac7Ac, Pse5Ac7Am, Pse8OAc, Pse5Am7AcGlnAc (AI151)] [Campylobacter jejuni subsp. jejuni NCTC 11168] | LytTR family two component transcriptional regulator | 1 | 0 | 0 | 0 | 0 | 0 | 0 | 0 | 0 | 0 | 0 | 0 | 0 | 0 | 0 | 0 | 0 | 0 | 0 | 1 |
| Fam41 | VFG011850(gi:15791455) | (flhG) | (flhG) ATP-binding protein [Flagella (CVF397)] [Campylobacter jejuni subsp. jejuni NCTC 11168] | conjugal transfer protein TraA | 16 | 1 | 2 | 0 | 0 | 0 | 0 | 0 | 0 | 2 | 2 | 2 | 2 | 2 | 0 | 0 | 1 | 0 | 2 | 0 |
| Fam78 | VFG013286(gi:16272302) | (galE) | (galE) UDP-glucose 4-epimerase [LOS (CVF494)] [Haemophilus influenzae Rd KW20] | UDP-glucose 4-epimerase | 19 | 1 | 1 | 1 | 1 | 1 | 1 | 1 | 1 | 1 | 1 | 1 | 1 | 1 | 1 | 1 | 1 | 1 | 1 | 1 |
| Fam17 | VFG000314(gi:15644988) | (gluE) | (gluE) UDP-glucose 4-epimerase [LPS (VF0056)] [Helicobacter pylori 26695] | UDP-N-acetylglucosamine 4-epimerase | 18 | 1 | 1 | 1 | 1 | 1 | 1 | 1 | 1 | 1 | 1 | 1 | 1 | 1 | 1 | 1 | 1 | 1 | 1 | 0 |
| Fam87 | VFG002365(gi:123443283) | (gmd) | (gmd) GDP-mannose 4,6-dehydratase [O-antigen (VF0392)] [Yersinia enterocolitica subsp. enterocolitica 8081] | GDP-D-mannose dehydratase | 7 | 0 | 0 | 0 | 0 | 0 | 0 | 0 | 0 | 1 | 1 | 1 | 1 | 1 | 0 | 0 | 0 | 0 | 2 | 0 |
| Fam94 | VFG002365(gi:123443283) | (gmd) | (gmd) GDP-mannose 4,6-dehydratase [O-antigen (VF0392)] [Yersinia enterocolitica subsp. enterocolitica 8081] | epimerase, partial | 1 | 1 | 0 | 0 | 0 | 0 | 0 | 0 | 0 | 0 | 0 | 0 | 0 | 0 | 0 | 0 | 0 | 0 | 0 | 0 |
| Fam99 | VFG000964(gi:15675935) | (hasC) | (hasC) UDP-glucose pyrophosphorylase [Hyaluronic acid capsule (VF0244)] [Streptococcus pyogenes M1 GAS] | UTP--glucose-1-phosphate uridylyltransferase | 1 | 0 | 0 | 0 | 1 | 0 | 0 | 0 | 0 | 0 | 0 | 0 | 0 | 0 | 0 | 0 | 0 | 0 | 0 | 0 |
| Fam123 | VFG000907(gi:26249408) | (hlyB) | (hlyB) Hemolysin B [Hemolysin (VF0225)] [Escherichia coli CFT073] | hypothetical protein | 1 | 0 | 0 | 0 | 0 | 0 | 0 | 0 | 0 | 0 | 0 | 0 | 0 | 0 | 0 | 0 | 0 | 0 | 1 | 0 |
| Fam54 | VFG000843(gi:75994496) | (hlyD) | (hlyD) hemolysin transport protein [Hemolysin (VF0207)] [Escherichia coli O157:H7 str. EDL933] | secretion protein HlyD | 7 | 1 | 1 | 0 | 0 | 0 | 0 | 0 | 0 | 0 | 0 | 0 | 0 | 0 | 1 | 1 | 1 | 0 | 1 | 1 |
| Fam12 | VFG001855(gi:52840925) | (htpB) | (htpB) Hsp60, 60K heat shock protein HtpB [Hsp60 (VF0159)] [Legionella pneumophila subsp. pneumophila str. Philadelphia 1] | molecular chaperone GroEL | 19 | 1 | 1 | 1 | 1 | 1 | 1 | 1 | 1 | 1 | 1 | 1 | 1 | 1 | 1 | 1 | 1 | 1 | 1 | 1 |
| Fam61 | VFG001284(gi:21284314) | (icaR) | (icaR) ica operon transcriptional regulator IcaR [Intercellular adhesion proteins (VF0014)] [Staphylococcus aureus subsp. aureus MW2] | TetR family transcriptional regulator | 19 | 1 | 1 | 1 | 1 | 1 | 1 | 1 | 1 | 1 | 1 | 1 | 1 | 1 | 1 | 1 | 1 | 1 | 1 | 1 |
| Fam7 | VFG001381(gi:57116734) | (icl) | (icl) Isocitrate lyase Icl (isocitrase) (isocitratase) [Isocitrate lyase (VF0253)] [Mycobacterium tuberculosis H37Rv] | isocitrate lyase | 19 | 1 | 1 | 1 | 1 | 1 | 1 | 1 | 1 | 1 | 1 | 1 | 1 | 1 | 1 | 1 | 1 | 1 | 1 | 1 |
| Fam69 | VFG045346(gi:37679074) | (IlpA) | (IlpA) immunogenic lipoprotein A [IlpA (VF0513)] [Vibrio vulnificus YJ016] | DL-methionine transporter subunit ; periplasmic-binding component of ABC superfamily | 19 | 1 | 1 | 1 | 1 | 1 | 1 | 1 | 1 | 1 | 1 | 1 | 1 | 1 | 1 | 1 | 1 | 1 | 1 | 1 |
| Fam60 | VFG012509(gi:26247127) | (iroC) | (iroC) ATP binding cassette transporter [Salmochelin (IA013)] [Escherichia coli CFT073] | ABC transporter ATP-binding protein | 19 | 1 | 1 | 1 | 1 | 1 | 1 | 1 | 1 | 1 | 1 | 1 | 1 | 1 | 1 | 1 | 1 | 1 | 1 | 1 |
| Fam16 | VFG009570(gi:15608488) | (irtA) | (irtA) Iron-regulated transporter IrtA [mycobactin (IA031)] [Mycobacterium tuberculosis H37Rv] | antibiotic ABC transporter ATP-binding protein | 19 | 1 | 1 | 1 | 1 | 1 | 1 | 1 | 1 | 1 | 1 | 1 | 1 | 1 | 1 | 1 | 1 | 1 | 1 | 1 |
| Fam44 | VFG037028(gi:15676142) | (katA) | (katA) catalase [KatA (VF0454)] [Neisseria meningitidis MC58] | catalase | 19 | 1 | 1 | 1 | 1 | 1 | 1 | 1 | 1 | 1 | 1 | 1 | 1 | 1 | 1 | 1 | 1 | 1 | 1 | 1 |
| Fam75 | VFG001861(gi:52840449) | (katA) | (katA) catalase/(hydro)peroxidase [KatAB (VF0168)] [Legionella pneumophila subsp. pneumophila str. Philadelphia 1] | catalase/hydroperoxidase HPI(I) | 19 | 1 | 1 | 1 | 1 | 1 | 1 | 1 | 1 | 1 | 1 | 1 | 1 | 1 | 1 | 1 | 1 | 1 | 1 | 1 |
| Fam113 | VFG037028(gi:15676142) | (katA) | (katA) catalase [KatA (VF0454)] | Catalase precursor | 1 | 0 | 0 | 0 | 0 | 0 | 0 | 0 | 0 | 0 | 0 | 0 | 0 | 0 | 0 | 0 | 0 | 1 | 0 | 0 |
| Fam32 | VFG013465(gi:16273457) | (kdsA) | (kdsA) 2-dehydro-3-deoxyphosphooctonate aldolase [LOS (CVF494)] [Haemophilus influenzae Rd KW20] | 2-dehydro-3-deoxyphosphogluconate aldolase | 19 | 1 | 1 | 1 | 1 | 1 | 1 | 1 | 1 | 1 | 1 | 1 | 1 | 1 | 1 | 1 | 1 | 1 | 1 | 1 |
| Fam14 | VFG000320(gi:15646084 | (kdtB) | (kdtB) lipopolysaccharide core biosynthesis protein [LPS (VF0056)] [Helicobacter pylori 26695] | phosphopantetheine adenylyltransferase | 19 | 1 | 1 | 1 | 1 | 1 | 1 | 1 | 1 | 1 | 1 | 1 | 1 | 1 | 1 | 1 | 1 | 1 | 1 | 1 |
| Fam84 | VFG013354(gi:16272808) | (kfiC) | (kfiC) lipopolysaccharide biosynthesis protein [LOS (CVF494)] [Haemophilus influenzae Rd KW20] | hypothetical protein | 9 | 1 | 1 | 1 | 1 | 0 | 0 | 0 | 0 | 1 | 1 | 1 | 1 | 1 | 0 | 0 | 0 | 0 | 0 | 0 |
| Fam121 | VFG013354(gi:16272808) | (kfiC) | (kfiC) lipopolysaccharide biosynthesis protein [LOS (CVF494)] [Haemophilus influenzae Rd KW20] | UDP-Glc:alpha-D-GlcNAc-diphosphoundecaprenol beta-1,3-glucosyltransferase WfgD | 1 | 0 | 0 | 0 | 0 | 0 | 0 | 0 | 0 | 0 | 0 | 0 | 0 | 0 | 0 | 0 | 0 | 1 | 0 | 0 |
| Fam124 | VFG013354(gi:16272808) | (kfiC) | (kfiC) lipopolysaccharide biosynthesis protein [LOS (CVF494)] [Haemophilus influenzae Rd KW20] | hypothetical protein | 1 | 0 | 0 | 0 | 0 | 0 | 0 | 0 | 0 | 0 | 0 | 0 | 0 | 0 | 0 | 0 | 0 | 0 | 1 | 0 |
| Fam31 | VFG001971(gi:15792761) | (kpsF) | (kpsF) D-arabinose 5-phosphate isomerase [Capsule (VF0323)] [Campylobacter jejuni subsp. jejuni NCTC 11168] | D-arabinose 5-phosphate isomerase | 19 | 1 | 1 | 1 | 1 | 1 | 1 | 1 | 1 | 1 | 1 | 1 | 1 | 1 | 1 | 1 | 1 | 1 | 1 | 1 |
| Fam5 | VFG001974(gi:15792764) | (kpsT) | (kpsT) capsule polysaccharide export ATP-binding protein [Capsule (VF0323)] [Campylobacter jejuni subsp. jejuni NCTC 11168] | glycosyl transferase family 2 | 19 | 1 | 1 | 1 | 1 | 1 | 1 | 1 | 1 | 1 | 1 | 1 | 1 | 1 | 1 | 1 | 1 | 1 | 1 | 1 |
| Fam6 | VFG001449(gi:146567) | (kpsT) | (kpsT) KpsT [K1 capsule (VF0239)] [Escherichia coli O18:K1:H7 str. RS218] | ABC transporter | 19 | 1 | 1 | 1 | 1 | 1 | 1 | 1 | 1 | 1 | 1 | 1 | 1 | 1 | 1 | 1 | 1 | 1 | 1 | 1 |
| Fam18 | VFG013471(gi:16273473) | (lgtA) | (lgtA) N-acetylglucosamine glycosyltransferase [LOS (CVF494)] [Haemophilus influenzae Rd KW20] | glycosyl transferase family A | 13 | 1 | 1 | 1 | 1 | 1 | 1 | 1 | 1 | 0 | 0 | 0 | 0 | 0 | 1 | 1 | 1 | 1 | 0 | 1 |
| Fam29 | VFG041304(gi:52842179) | (lirB) | (lirB) Dot/Icm type IV secretion system effector LirB [Dot/Icm (SS047)] [Legionella pneumophila subsp. pneumophila str. Philadelphia 1] | peptidylprolyl isomerase | 19 | 1 | 1 | 1 | 1 | 1 | 1 | 1 | 1 | 1 | 1 | 1 | 1 | 1 | 1 | 1 | 1 | 1 | 1 | 1 |
| Fam40 | VFG013390(gi:16272992) | (lpxA) | (lpxA) UDP-N-acetylglucosamine acyltransferase [LOS (CVF494)] [Haemophilus influenzae Rd KW20] | UDP-N-acetylglucosamine acyltransferase | 19 | 1 | 1 | 1 | 1 | 1 | 1 | 1 | 1 | 1 | 1 | 1 | 1 | 1 | 1 | 1 | 1 | 1 | 1 | 1 |
| Fam93 | VFG013487(gi:16273583) | (lsgE) | (lsgE) glycosyltransferase [LOS (CVF494)] [Haemophilus influenzae Rd KW20] | hypothetical protein | 4 | 1 | 1 | 0 | 0 | 0 | 0 | 1 | 1 | 0 | 0 | 0 | 0 | 0 | 0 | 0 | 0 | 0 | 0 | 0 |
| Fam42 | VFG000574(gi:16767047) | (mgtB) | (mgtB) Mg2+ transport protein [MgtBC (VF0106)] [Salmonella enterica subsp. enterica serovar Typhimurium str. LT2] | ATPase | 19 | 1 | 1 | 1 | 1 | 1 | 1 | 1 | 1 | 1 | 1 | 1 | 1 | 1 | 1 | 1 | 1 | 1 | 1 | 1 |
| Fam39 | VFG000574(gi:16767047) | (mgtB) | (mgtB) Mg2+ transport protein [MgtBC (VF0106)] [Salmonella enterica subsp. enterica serovar Typhimurium str. LT2] | magnesium ABC transporter ATPase | 19 | 1 | 1 | 1 | 1 | 1 | 1 | 1 | 1 | 1 | 1 | 1 | 1 | 1 | 1 | 1 | 1 | 1 | 1 | 1 |
| Fam38 | VFG001395(gi:15608948) | (mgtC) | (mgtC) Possible Mg2+ transport P-type ATPase C MgtC [MgtC (VF0289)] [Mycobacterium tuberculosis H37Rv] | magnesium transporter MgtC | 19 | 1 | 1 | 1 | 1 | 1 | 1 | 1 | 1 | 1 | 1 | 1 | 1 | 1 | 1 | 1 | 1 | 1 | 1 | 1 |
| Fam104 | VFG001395(gi:15608948) | (mgtC) | (mgtC) Possible Mg2+ transport P-type ATPase C MgtC [MgtC (VF0289)] [Mycobacterium tuberculosis H37Rv] | MgtC/SapB transporter | 2 | 0 | 0 | 0 | 0 | 0 | 0 | 0 | 0 | 0 | 0 | 0 | 0 | 0 | 0 | 0 | 0 | 0 | 1 | 1 |
| Fam33 | VFG001864(gi:52841028) | (mip) | (mip) macrophage infectivity potentiator Mip [Mip (VF0153)] [Legionella pneumophila subsp. pneumophila str. Philadelphia 1] | peptidylprolyl isomerase | 18 | 1 | 1 | 1 | 1 | 1 | 1 | 1 | 1 | 1 | 1 | 1 | 1 | 1 | 1 | 1 | 1 | 1 | 0 | 1 |
| Fam97 | VFG013248(gi:16272034) | (msbA) | (msbA) lipid transporter ATP-binding/permease [LOS (CVF494)] [Haemophilus influenzae Rd KW20] | bacteriocin/lantibiotic ABC transporter ATP-binding protein | 2 | 0 | 0 | 0 | 0 | 2 | 0 | 0 | 0 | 0 | 0 | 0 | 0 | 0 | 0 | 0 | 0 | 0 | 0 | 0 |
| Fam37 | VFG037100(gi:15675984) | (msrA/B(pilB) | (msrA/B(pilB)) trifunctional thioredoxin/methionine sulfoxide reductase A/B protein [MsrAB (VF0456)] [Neisseria meningitidis MC58] | methionine-R-sulfoxide reductase | 37 | 2 | 2 | 2 | 2 | 2 | 2 | 2 | 2 | 2 | 2 | 2 | 2 | 2 | 2 | 2 | 2 | 1 | 2 | 2 |
| Fam48 | VFG037100(gi:15675984) | (msrA/B(pilB)) | (msrA/B(pilB)) trifunctional thioredoxin/methionine sulfoxide reductase A/B protein [MsrAB (VF0456)] [Neisseria meningitidis MC58] | Peptide methionine sulfoxide reductase MsrA | 19 | 1 | 1 | 1 | 1 | 1 | 1 | 1 | 1 | 1 | 1 | 1 | 1 | 1 | 1 | 1 | 1 | 1 | 1 | 1 |
| Fam64 | VFG037100(gi:15675984) | (msrA/B(pilB)) | (msrA/B(pilB)) trifunctional thioredoxin/methionine sulfoxide reductase A/B protein [MsrAB (VF0456)] [Neisseria meningitidis MC58] | Peptide methionine sulfoxide reductase MsrA / Peptide methionine sulfoxide reductase MsrB | 19 | 1 | 1 | 1 | 1 | 1 | 1 | 1 | 1 | 1 | 1 | 1 | 1 | 1 | 1 | 1 | 1 | 1 | 1 | 1 |
| Fam107 | VFG002283(gi:18309707) | (nanI) | (nanI) exo-alpha-sialidase [sialidase (VF0391)] [Clostridium perfringens str. 13] | secreted sialidase | 2 | 0 | 0 | 0 | 0 | 0 | 0 | 0 | 0 | 0 | 0 | 0 | 0 | 0 | 0 | 0 | 0 | 0 | 1 | 1 |
| Fam23 | VFG001338(gi:22537319) | (neuB) | (neuB) N-acetyl neuramic acid synthetase NeuB [Capsule (VF0274)] [Streptococcus agalactiae 2603V/R] | hypothetical protein C874_10245 | 1 | 0 | 0 | 0 | 0 | 0 | 0 | 0 | 0 | 0 | 0 | 0 | 0 | 0 | 0 | 0 | 1 | 0 | 0 | 0 |
| Fam10 | VFG013265(gi:16272217) | (orfM) | (orfM) deoxyribonucleotide triphosphate pyrophosphatase [LOS (CVF494)] [Haemophilus influenzae Rd KW20] | deoxyribonucleotide triphosphate pyrophosphatase | 19 | 1 | 1 | 1 | 1 | 1 | 1 | 1 | 1 | 1 | 1 | 1 | 1 | 1 | 1 | 1 | 1 | 1 | 1 | 1 |
| Fam98 | VFG013265(gi:16272217) | (orfM) | (orfM) deoxyribonucleotide triphosphate pyrophosphatase [LOS (CVF494)] [Haemophilus influenzae Rd KW20] | Nucleoside 5-triphosphatase RdgB (dHAPTP, dITP, XTP-specific) | 1 | 0 | 0 | 0 | 0 | 1 | 0 | 0 | 0 | 0 | 0 | 0 | 0 | 0 | 0 | 0 | 0 | 0 | 0 | 0 |
| Fam34 | VFG001354(gi:15900810) | (pce) | (pce) choline binding protein E [CBPs (VF0145)] [Streptococcus pneumoniae TIGR4] | beta-lactamase | 19 | 1 | 1 | 1 | 1 | 1 | 1 | 1 | 1 | 1 | 1 | 1 | 1 | 1 | 1 | 1 | 1 | 1 | 1 | 1 |
| Fam57 | VFG000165(gi:15599421) | (pchF) | (pchF) pyochelin synthetase PchF [Pyochelin (VF0095)] [Pseudomonas aeruginosa PAO1] | AMP-dependent synthetase | 19 | 1 | 1 | 1 | 1 | 1 | 1 | 1 | 1 | 1 | 1 | 1 | 1 | 1 | 1 | 1 | 1 | 1 | 1 | 1 |
| Fam74 | VFG000167(gi:15599423) | (pchR) | (pchR) transcriptional regulator PchR [Pyochelin (VF0095)] [Pseudomonas aeruginosa PAO1] | Transcription activator, effector binding protein | 19 | 1 | 1 | 1 | 1 | 1 | 1 | 1 | 1 | 1 | 1 | 1 | 1 | 1 | 1 | 1 | 1 | 1 | 1 | 1 |
| Fam112 | VFG001225(gi:15595605 | (pilG) | (pilG) twitching motility protein PilG [Type IV pili (VF0082)] [Pseudomonas aeruginosa PAO1] | Transcriptional regulatory protein YycF | 1 | 0 | 0 | 0 | 0 | 0 | 0 | 0 | 0 | 0 | 0 | 0 | 0 | 0 | 0 | 0 | 0 | 1 | 0 | 0 |
| Fam56 | VFG001214(gi:15599743) | (pilR) | (pilR) two-component response regulator PilR [Type IV pili (VF0082)] [Pseudomonas aeruginosa PAO1] | Fis family transcriptional regulator | 19 | 1 | 1 | 1 | 1 | 1 | 1 | 1 | 1 | 1 | 1 | 1 | 1 | 1 | 1 | 1 | 1 | 1 | 1 | 1 |
| Fam58 | VFG002538(gi:53721911) | (pmlR/bspR1) | (pmlR/bspR1) N-acylhomoserine lactone dependent regulatory protein [Quorum-sensing (VF0433)] [Burkholderia pseudomallei K96243] | LuxR family transcriptional regulator | 19 | 1 | 1 | 1 | 1 | 1 | 1 | 1 | 1 | 1 | 1 | 1 | 1 | 1 | 1 | 1 | 1 | 1 | 1 | 1 |
| Fam102 | VFG002538(gi:53721911) | (pmlR/bspR1) | (pmlR/bspR1) N-acylhomoserine lactone dependent regulatory protein [Quorum-sensing (VF0433)] [Burkholderia pseudomallei K96243] | LuxR family transcriptional regulator | 1 | 0 | 0 | 0 | 0 | 0 | 0 | 0 | 0 | 0 | 0 | 0 | 0 | 0 | 0 | 0 | 0 | 0 | 0 | 1 |
| Fam67 | VFG032878(gi:16804258) | (prsA2) | (prsA2) post translocation chaperone PrsA2 [PrsA2 (VF0449)] [Listeria monocytogenes EGD-e] | PpiC-type peptidyl-prolyl cis-trans isomerase | 19 | 1 | 1 | 1 | 1 | 1 | 1 | 1 | 1 | 1 | 1 | 1 | 1 | 1 | 1 | 1 | 1 | 1 | 1 | 1 |
| Fam15 | VFG002374(gi:123443292) | (prt) | (prt) paratose synthase [O-antigen (VF0392)] [Yersinia enterocolitica subsp. enterocolitica 8081] | GDP-mannose 4,6-dehydratase | 17 | 1 | 1 | 1 | 1 | 1 | 1 | 1 | 1 | 1 | 1 | 1 | 1 | 1 | 1 | 1 | 1 | 1 | 0 | 0 |
| Fam25 | VFG001921(gi:15792654) | (ptmB) | (ptmB) acylneuraminate cytidylyltransferase [Flagella (VF0114)] [Campylobacter jejuni subsp. jejuni NCTC 11168] | hypothetical protein C874_10260 | 1 | 0 | 0 | 0 | 0 | 0 | 0 | 0 | 0 | 0 | 0 | 0 | 0 | 0 | 0 | 0 | 1 | 0 | 0 | 0 |
| Fam62 | VFG000160(gi:15597593) | (pvdE) | (pvdE) pyoverdine biosynthesis protein PvdE [Pyoverdine (VF0094)] [Pseudomonas aeruginosa PAO1] | ABC transporter ATP-binding protein yadG | 19 | 1 | 1 | 1 | 1 | 1 | 1 | 1 | 1 | 1 | 1 | 1 | 1 | 1 | 1 | 1 | 1 | 1 | 1 | 1 |
| Fam96 | VFG000315(gi:15644674) | (rfbM) | (rfbM) mannose-6-phosphate isomerase [LPS (VF0056)] [Helicobacter pylori 26695] | Mannose-1-phosphate guanylyltransferase/mannose-6-phosphate isomerase | 2 | 0 | 0 | 1 | 1 | 0 | 0 | 0 | 0 | 0 | 0 | 0 | 0 | 0 | 0 | 0 | 0 | 0 | 0 | 0 |
| Fam28 | VFG013368(gi:30995408) | (rffG) | (rffG) dTDP-glucose 46-dehydratase [LOS (CVF494)] [Haemophilus influenzae Rd KW20] | dTDP-glucose 4,6-dehydratase | 19 | 1 | 1 | 1 | 1 | 1 | 1 | 1 | 1 | 1 | 1 | 1 | 1 | 1 | 1 | 1 | 1 | 1 | 1 | 1 |
| Fam109 | VFG045340(gi:17987019) | (ricA) | (ricA) Rab2 interacting conserved protein A [RicA (VF0414)] [Brucella melitensis bv. 1 str. 16M] | hypothetical protein | 1 | 0 | 0 | 0 | 0 | 0 | 0 | 0 | 0 | 0 | 0 | 0 | 0 | 0 | 0 | 0 | 0 | 0 | 0 | 1 |
| Fam122 | VFG000674(gi:6006577) | (smcL) | (smcL) sphingomyelinase-c [SMase (VF0061)] [Listeria ivanovii str. ATCC 19119] | Sphingomyelinase C precursor | 2 | 0 | 0 | 0 | 0 | 0 | 0 | 0 | 0 | 0 | 0 | 0 | 0 | 0 | 0 | 0 | 0 | 1 | 1 | 0 |
| Fam8 | VFG001867(gi:52843161) | (sodB) | (sodB) superoxide dismutase [SodB (VF0169)] [Legionella pneumophila subsp. pneumophila str. Philadelphia 1] | superoxide dismutase [ | 19 | 1 | 1 | 1 | 1 | 1 | 1 | 1 | 1 | 1 | 1 | 1 | 1 | 1 | 1 | 1 | 1 | 1 | 1 | 1 |
| Fam43 | VFG001867(gi:52843161) | (sodB) | (sodB) superoxide dismutase [SodB (VF0169)] [Legionella pneumophila subsp. pneumophila str. Philadelphia 1] | superoxide dismutase | 19 | 1 | 1 | 1 | 1 | 1 | 1 | 1 | 1 | 1 | 1 | 1 | 1 | 1 | 1 | 1 | 1 | 1 | 1 | 1 |
| Fam73 | VFG001867(gi:52843161) | (sodB) | (sodB) superoxide dismutase [SodB (VF0169)] [Legionella pneumophila subsp. pneumophila str. Philadelphia 1] | superoxide dismutase (Mn) | 19 | 1 | 1 | 1 | 1 | 1 | 1 | 1 | 1 | 1 | 1 | 1 | 1 | 1 | 1 | 1 | 1 | 1 | 1 | 1 |
| Fam9 | VFG002059(gi:15595271) | (tagT) | (tagT) type six secretion associated protein TagT, ATP-binding component of ABC transporter [HSI-I (VF0334)] [Pseudomonas aeruginosa PAO1] | ABC transporter ATP-binding protein | 19 | 1 | 1 | 1 | 1 | 1 | 1 | 1 | 1 | 1 | 1 | 1 | 1 | 1 | 1 | 1 | 1 | 1 | 1 | 1 |
| Fam19 | VFG002059(gi:15595271) | (tagT) | (tagT) type six secretion associated protein TagT, ATP-binding component of ABC transporter [HSI-I (VF0334)] [Pseudomonas aeruginosa PAO1] | ABC transporter | 19 | 1 | 1 | 1 | 1 | 1 | 1 | 1 | 1 | 1 | 1 | 1 | 1 | 1 | 1 | 1 | 1 | 1 | 1 | 1 |
| Fam76 | VFG000101(gi:15640855) | (tcpN/toxT) | (tcpN/toxT) TCP pilus virulence regulatory protein [TCP (VF0126)] [Vibrio cholerae O1 biovar El Tor str. N16961] | AraC family transcriptional regulator | 19 | 1 | 1 | 1 | 1 | 1 | 1 | 1 | 1 | 1 | 1 | 1 | 1 | 1 | 1 | 1 | 1 | 1 | 1 | 1 |
| Fam72 | VFG002480(gi:53722524) | (tssH-5/clpV) | (tssH-5/clpV) Clp-type ATPase chaperone protein [T6SS-1 (VF0429)] [Burkholderia pseudomallei K96243] | ATPase AAA | 19 | 1 | 1 | 1 | 1 | 1 | 1 | 1 | 1 | 1 | 1 | 1 | 1 | 1 | 1 | 1 | 1 | 1 | 1 | 1 |
| Fam2 | VFG000431(gi:16763123) | (tviB) | (tviB) Vi polysaccharide biosynthesis protein, UDP-glucose/GDP-mannose dehydrogenase TviB [Vi antigen (VF0101)] [Salmonella enterica subsp. enterica serovar Typhi str. CT18] | Vi polysaccharide biosynthesis protein VipA/TviB | 21 | 1 | 1 | 1 | 1 | 1 | 1 | 1 | 1 | 1 | 1 | 1 | 1 | 1 | 1 | 2 | 1 | 2 | 1 | 1 |
| Fam100 | VFG000430(gi:16763122) | (tviC) | (tviC) Vi polysaccharide biosynthesis protein, epimerase TviC [Vi antigen (VF0101)] [Salmonella enterica subsp. enterica serovar Typhi str. CT18] | Vi polysaccharide biosynthesis protein VipB/TviC [ | 2 | 0 | 0 | 0 | 0 | 0 | 0 | 0 | 0 | 0 | 0 | 0 | 0 | 0 | 0 | 1 | 0 | 1 | 0 | 0 |
| Fam117 | VFG000269(gi:15644703) | (ureA) | (ureA) urease alpha subunit UreA [Urease (VF0050)] [Helicobacter pylori 26695] | Urease subunit beta | 2 | 0 | 0 | 0 | 0 | 0 | 0 | 0 | 0 | 0 | 0 | 0 | 0 | 0 | 0 | 0 | 0 | 1 | 1 | 0 |
| Fam118 | VFG000269(gi:15644703 | (ureA) | (ureA) urease alpha subunit UreA [Urease (VF0050)] [Helicobacter pylori 26695] | Urease subunit gamma | 2 | 0 | 0 | 0 | 0 | 0 | 0 | 0 | 0 | 0 | 0 | 0 | 0 | 0 | 0 | 0 | 0 | 1 | 1 | 0 |
| Fam116 | VFG000270(gi:15644702) | (ureB) | (ureB) urease beta subunit UreB, urea amidohydrolase [Urease (VF0050)] [Helicobacter pylori 26695] | Urease subunit alpha | 2 | 0 | 0 | 0 | 0 | 0 | 0 | 0 | 0 | 0 | 0 | 0 | 0 | 0 | 0 | 0 | 0 | 1 | 1 | 0 |
| Fam115 | VFG000272(gi:15644700) | (ureE) | (ureE) urease accessory protein (ureE) [Urease (VF0050)] [Helicobacter pylori 26695] | Urease accessory protein UreE | 2 | 0 | 0 | 0 | 0 | 0 | 0 | 0 | 0 | 0 | 0 | 0 | 0 | 0 | 0 | 0 | 0 | 1 | 1 | 0 |
| Fam114 | VFG000274(gi:15644698) | (ureG) | (ureG) urease accessory protein (ureG) [Urease (VF0050)] [Helicobacter pylori 26695] | Urease accessory protein UreG | 2 | 0 | 0 | 0 | 0 | 0 | 0 | 0 | 0 | 0 | 0 | 0 | 0 | 0 | 0 | 0 | 0 | 1 | 1 | 0 |
| Fam4 | VFG002085(gi:15600888) | (vasH) | (vasH) type VI secretion system regulatory protein VasH [T6SS (VF0335)] [Vibrio cholerae O1 biovar El Tor str. N16961] | ATPase AAA | 19 | 1 | 1 | 1 | 1 | 1 | 1 | 1 | 1 | 1 | 1 | 1 | 1 | 1 | 1 | 1 | 1 | 1 | 1 | 1 |
| Fam59 | VFG010763(gi:52841459) | (vpdB) | (vpdB) Dot/Icm type IV secretion system effector VpdB [Dot/Icm (SS047)] [Legionella pneumophila subsp. pneumophila str. Philadelphia 1] | Patatin | 19 | 1 | 1 | 1 | 1 | 1 | 1 | 1 | 1 | 1 | 1 | 1 | 1 | 1 | 1 | 1 | 1 | 1 | 1 | 1 |
| Fam11 | VFG002563(gi:53720413) | (wzt2) | (wzt2) ATP-binding ABC transporter capsular polysaccharide export protein [Capsule I (VF0436)] [Burkholderia pseudomallei K96243] | ATPase component of ABC transporter with duplicated ATPase domains | 18 | 1 | 1 | 1 | 1 | 1 | 0 | 1 | 1 | 1 | 1 | 1 | 1 | 1 | 1 | 1 | 1 | 1 | 1 | 1 |
| Fam51 | VFG000366(gi:16122162) | (ybtQ) | (ybtQ) inner membrane ABC-transporter YbtQ [Yersiniabactin (VF0136)] [Yersinia pestis CO92] | antibiotic ABC transporter ATP-binding protein | 19 | 1 | 1 | 1 | 1 | 1 | 1 | 1 | 1 | 1 | 1 | 1 | 1 | 1 | 1 | 1 | 1 | 1 | 1 | 1 |
| Fam80 | VFG000366(gi:16122162) | (ybtQ) | (ybtQ) inner membrane ABC-transporter YbtQ [Yersiniabactin (VF0136)] [Yersinia pestis CO92] | ABC transporter-like protein | 1 | 0 | 0 | 0 | 0 | 0 | 1 | 0 | 0 | 0 | 0 | 0 | 0 | 0 | 0 | 0 | 0 | 0 | 0 | 0 |
| Total No. of proteins per genome: | | | |  |  | 79 | 79 | 77 | 78 | 78 | 75 | 75 | 75 | 81 | 81 | 81 | 81 | 81 | 73 | 78 | 79 | 83 | 88 | 80 |
| # Virulence Factor database identifier | | | |  |  |  |  |  |  |  |  |  |  |  |  |  |  |  |  |  |  |  |  |  |
|  |  |  |  |  |  |  |  |  |  |  |  |  |  |  |  |  |  |  |  |  |  |  |  |  |

| **Table S5. Secretion systems predicted in *Elizabethkingia* genomes using MacSyFinder # £** | | | | | | | | | | | | | |  |  |  |  |  |  |  |  |  |
| --- | --- | --- | --- | --- | --- | --- | --- | --- | --- | --- | --- | --- | --- | --- | --- | --- | --- | --- | --- | --- | --- | --- |
|  |  |  |  | ***E. anophelis*** | |  |  |  |  |  |  |  |  |  |  |  |  |  |  | ***E. miricola*** | | ***E. meningoseptica*** |
|  |  |  | Strain name: | NUH6 | NUH11 | E27107 | E18064 | R26 | Ag1 | PW2809 | PW2806 | NUH1 | NUH4 | NUHP1 | NUHP3 | NUHP2 | PW2810 | B2D | 502 | BM10 | ATCC33958 | NBRC1235 |
|  |  |  | Study id: | ELAN006 | ELAN004 | ELAN013 | ELAN015 | ELAN014 | ELAN002 | ELAN010 | ELAN011 | ELAN003 | ELAN005 | ELAN007 | ELAN009 | ELAN008 | ELAN012 | ELAN016 | ELAN001 | ELMI001 | ELMI002 | ELME001 |
|  |  |  | **T1SS** | 1 | 1 | 1 | 1 | 2 | 2 | 1 | 1 | 1 | 1 | 1 | 1 | 1 | 2 | 2 | 1 | 2 | 2 | 1 |
|  |  |  | **T4SS_B** | 0 | 1 | 1 | 1 | 2 | 2 | 1 | 1 | 4 | 3 | 4 | 4 | 4 | 0 | 0 | 1 | 1 | 4 | 0 |
|  |  |  | **T6SSiii** | 1 | 1 | 1 | 1 | 1 | 1 | 1 | 1 | 1 | 1 | 1 | 1 | 1 | 1 | 1 | 1 | 1 | 1 | 1 |
|  |  |  |  |  |  |  |  |  |  |  |  |  |  |  |  |  |  |  |  |  |  |  |
| # Please see http://macsyfinder.readthedocs.org/en/latest/system_definition.html | | | | | | | | | | | | |  |  |  |  |  |  |  |  |  |  |
|  |  |  |  |  |  |  |  |  |  |  |  |  |  |  |  |  |  |  |  |  |  |  |
| **£ Raw results: Gene families matching HMM protein profiles from bacterial secretion systems** | | | | | | | | | | | | | | |  |  |  |  |  |  |  |  |
|  |  |  |  | ***E. anophelis*** | |  |  |  |  |  |  |  |  |  |  |  |  |  |  | ***E. miricola*** | | ***E. meningoseptica*** |
|  |  |  |  | NUH6 | NUH11 | E27107 | E18064 | R26 | Ag1 | PW2809 | PW2806 | NUH1 | NUH4 | NUHP1 | NUHP3 | NUHP2 | PW2810 | B2D | 502 | BM10 | ATCC33958 | NBRC1235 |
| Familly | HMM_profiles_Macsyfinder | TSS-Type | VGF-Name | ELAN006 | ELAN004 | ELAN013 | ELAN015 | ELAN014 | ELAN002 | ELAN010 | ELAN011 | ELAN003 | ELAN005 | ELAN007 | ELAN009 | ELAN008 | ELAN012 | ELAN016 | ELAN001 | ELMI001 | ELMI002 | ELME001 |
| Fam16 | T1SS_abc | T1SS |  | 1 | 1 | 1 | 1 | 1 | 1 | 2 | 2 | 1 | 1 | 1 | 1 | 1 | 1 | 1 | 1 | 2 | 1 | 1 |
| Fam37 | T1SS_abc | T1SS |  | 0 | 0 | 0 | 0 | 0 | 1 | 0 | 0 | 0 | 0 | 0 | 0 | 0 | 1 | 1 | 0 | 0 | 1 | 0 |
| Fam14 | T1SS_mfp | T1SS | VFG000843(gi:75994496) | 1 | 1 | 0 | 0 | 0 | 0 | 0 | 0 | 0 | 0 | 0 | 0 | 0 | 1 | 1 | 1 | 0 | 1 | 1 |
| Fam17 | T1SS_mfp | T1SS |  | 0 | 0 | 1 | 1 | 1 | 1 | 1 | 1 | 1 | 1 | 1 | 1 | 1 | 1 | 1 | 1 | 2 | 1 | 1 |
| Fam38 | T1SS_mfp | T1SS |  | 0 | 0 | 0 | 0 | 1 | 1 | 0 | 0 | 0 | 0 | 0 | 0 | 0 | 1 | 1 | 0 | 0 | 1 | 0 |
| Fam15 | T1SS_omf | T1SS |  | 0 | 0 | 1 | 1 | 1 | 1 | 1 | 1 | 1 | 1 | 1 | 1 | 1 | 1 | 1 | 1 | 1 | 1 | 1 |
| Fam11 | T4SS_B_traE | T4SS_B |  | 1 | 2 | 0 | 0 | 0 | 0 | 1 | 1 | 2 | 2 | 2 | 2 | 2 | 0 | 0 | 1 | 0 | 3 | 0 |
| Fam33 | T4SS_B_traE | T4SS_B |  | 3 | 1 | 1 | 1 | 1 | 2 | 0 | 0 | 4 | 3 | 4 | 4 | 4 | 0 | 0 | 0 | 1 | 1 | 0 |
| Fam10 | T4SS_B_traF | T4SS_B |  | 1 | 2 | 0 | 0 | 0 | 0 | 1 | 1 | 2 | 2 | 1 | 2 | 2 | 0 | 0 | 1 | 0 | 3 | 0 |
| Fam50 | T4SS_B_traI | T4SS_B |  | 0 | 0 | 0 | 0 | 1 | 0 | 0 | 0 | 0 | 0 | 0 | 0 | 0 | 0 | 0 | 0 | 0 | 0 | 0 |
| Fam8 | T4SS_B_traI | T4SS_B |  | 1 | 2 | 0 | 0 | 0 | 0 | 1 | 1 | 2 | 2 | 2 | 2 | 2 | 0 | 0 | 1 | 0 | 3 | 0 |
| Fam30 | T4SS_B_traJ | T4SS_B |  | 3 | 1 | 1 | 1 | 2 | 2 | 0 | 0 | 4 | 3 | 4 | 4 | 4 | 0 | 0 | 0 | 1 | 1 | 0 |
| Fam7 | T4SS_B_traJ | T4SS_B |  | 1 | 2 | 0 | 0 | 0 | 0 | 1 | 1 | 2 | 2 | 2 | 2 | 2 | 0 | 0 | 1 | 0 | 3 | 0 |
| Fam29 | T4SS_B_traK | T4SS_B |  | 3 | 1 | 1 | 1 | 1 | 2 | 0 | 0 | 3 | 3 | 4 | 3 | 3 | 0 | 0 | 0 | 1 | 1 | 0 |
| Fam51 | T4SS_B_traK | T4SS_B |  | 0 | 0 | 0 | 0 | 1 | 0 | 0 | 0 | 0 | 0 | 0 | 0 | 0 | 0 | 0 | 0 | 0 | 0 | 0 |
| Fam6 | T4SS_B_traK | T4SS_B |  | 1 | 2 | 0 | 0 | 0 | 0 | 1 | 1 | 2 | 2 | 2 | 2 | 2 | 0 | 0 | 1 | 0 | 3 | 0 |
| Fam40 | T4SS_B_traM | T4SS_B |  | 1 | 1 | 0 | 0 | 0 | 0 | 1 | 1 | 2 | 2 | 2 | 2 | 2 | 0 | 0 | 0 | 0 | 3 | 0 |
| Fam44 | T4SS_B_traM | T4SS_B |  | 2 | 1 | 1 | 1 | 0 | 0 | 0 | 0 | 3 | 2 | 3 | 3 | 3 | 0 | 0 | 0 | 0 | 1 | 0 |
| Fam5 | T4SS_B_traM | T4SS_B |  | 0 | 1 | 0 | 0 | 0 | 0 | 0 | 0 | 0 | 0 | 0 | 0 | 0 | 0 | 0 | 1 | 0 | 0 | 0 |
| Fam35 | T4SS_B_traN | T4SS_B |  | 3 | 1 | 1 | 1 | 1 | 1 | 0 | 0 | 4 | 3 | 4 | 4 | 4 | 0 | 0 | 0 | 1 | 1 | 0 |
| Fam4 | T4SS_B_traN | T4SS_B |  | 0 | 1 | 0 | 0 | 0 | 0 | 0 | 0 | 0 | 0 | 0 | 0 | 0 | 0 | 0 | 1 | 0 | 0 | 0 |
| Fam41 | T4SS_B_traN | T4SS_B |  | 1 | 1 | 0 | 0 | 0 | 0 | 1 | 1 | 2 | 2 | 2 | 2 | 2 | 0 | 0 | 0 | 0 | 3 | 0 |
| Fam42 | T4SS_B_traO | T4SS_B |  | 1 | 1 | 0 | 0 | 0 | 0 | 1 | 1 | 2 | 2 | 2 | 2 | 2 | 0 | 0 | 0 | 0 | 3 | 0 |
| Fam31 | T4SS_B_traP | T4SS_B |  | 3 | 0 | 1 | 1 | 2 | 2 | 0 | 0 | 4 | 3 | 4 | 4 | 4 | 0 | 0 | 0 | 0 | 1 | 0 |
| Fam39 | T4SS_B_traP | T4SS_B |  | 1 | 0 | 1 | 1 | 0 | 0 | 0 | 0 | 2 | 2 | 2 | 2 | 2 | 0 | 0 | 0 | 0 | 1 | 0 |
| Fam53 | T4SS_B_traP | T4SS_B |  | 0 | 0 | 0 | 0 | 0 | 0 | 0 | 0 | 0 | 0 | 0 | 0 | 0 | 0 | 0 | 0 | 1 | 0 | 0 |
| Fam43 | T4SS_B_traQ | T4SS_B |  | 1 | 1 | 0 | 0 | 0 | 0 | 1 | 1 | 1 | 1 | 1 | 1 | 1 | 0 | 0 | 0 | 0 | 3 | 0 |
| Fam45 | T4SS_B_traQ | T4SS_B |  | 0 | 0 | 0 | 0 | 0 | 0 | 0 | 0 | 1 | 1 | 1 | 1 | 1 | 0 | 0 | 0 | 0 | 0 | 0 |
| Fam46 | T4SS_B_traQ | T4SS_B |  | 0 | 0 | 0 | 0 | 1 | 0 | 0 | 0 | 0 | 0 | 0 | 0 | 0 | 0 | 0 | 0 | 0 | 0 | 0 |
| Fam47 | T4SS_MOBB | T4SS_B |  | 0 | 0 | 0 | 0 | 1 | 0 | 0 | 0 | 0 | 0 | 0 | 0 | 0 | 0 | 0 | 0 | 0 | 0 | 0 |
| Fam12 | T4SS_MOBP1 | T4SS_B |  | 1 | 2 | 0 | 0 | 0 | 0 | 1 | 1 | 2 | 2 | 2 | 2 | 2 | 0 | 0 | 1 | 0 | 3 | 0 |
| Fam36 | T4SS_MOBP1 | T4SS_B |  | 3 | 1 | 1 | 1 | 2 | 2 | 0 | 0 | 4 | 3 | 4 | 4 | 4 | 0 | 0 | 0 | 1 | 1 | 0 |
| Fam52 | T4SS_t4cp1 | T4SS_B |  | 0 | 0 | 0 | 0 | 1 | 0 | 0 | 0 | 0 | 0 | 0 | 0 | 0 | 0 | 0 | 0 | 0 | 0 | 0 |
| Fam13 | T4SS_t4cp2 | T4SS_B |  | 1 | 2 | 0 | 0 | 0 | 0 | 1 | 1 | 2 | 2 | 2 | 2 | 2 | 0 | 0 | 1 | 0 | 3 | 0 |
| Fam32 | T4SS_t4cp2 | T4SS_B |  | 3 | 1 | 1 | 1 | 1 | 2 | 0 | 0 | 4 | 3 | 4 | 4 | 4 | 0 | 0 | 0 | 1 | 1 | 0 |
| Fam48 | T4SS_t4cp2 | T4SS_B |  | 0 | 0 | 0 | 0 | 1 | 0 | 0 | 0 | 0 | 0 | 0 | 0 | 0 | 0 | 0 | 0 | 0 | 0 | 0 |
| Fam34 | T4SS_virb4 | T4SS_B |  | 3 | 1 | 1 | 1 | 1 | 2 | 0 | 0 | 4 | 3 | 4 | 4 | 4 | 0 | 0 | 0 | 1 | 1 | 0 |
| Fam9 | T4SS_virb4 | T4SS_B |  | 1 | 2 | 0 | 0 | 0 | 0 | 1 | 1 | 2 | 2 | 2 | 2 | 2 | 0 | 0 | 1 | 0 | 3 | 0 |
| Fam25 | T6SSiii_tssB | T6SSiii |  | 1 | 1 | 1 | 1 | 1 | 1 | 1 | 1 | 1 | 1 | 1 | 1 | 1 | 1 | 1 | 1 | 1 | 1 | 1 |
| Fam26 | T6SSiii_tssC | T6SSiii |  | 1 | 1 | 1 | 1 | 1 | 1 | 1 | 1 | 1 | 1 | 1 | 1 | 1 | 1 | 1 | 1 | 1 | 1 | 1 |
| Fam2 | T6SSiii_tssD | T6SSiii |  | 1 | 1 | 1 | 1 | 1 | 1 | 1 | 1 | 1 | 1 | 1 | 1 | 1 | 1 | 1 | 1 | 1 | 1 | 1 |
| Fam20 | T6SSiii_tssE | T6SSiii |  | 1 | 1 | 1 | 1 | 1 | 1 | 1 | 1 | 1 | 1 | 1 | 1 | 1 | 1 | 1 | 1 | 1 | 1 | 1 |
| Fam21 | T6SSiii_tssF | T6SSiii |  | 1 | 1 | 1 | 1 | 1 | 1 | 1 | 1 | 1 | 1 | 1 | 1 | 1 | 1 | 1 | 1 | 1 | 1 | 1 |
| Fam22 | T6SSiii_tssG | T6SSiii |  | 1 | 1 | 1 | 1 | 1 | 1 | 1 | 1 | 1 | 1 | 1 | 1 | 1 | 1 | 1 | 1 | 1 | 1 | 1 |
| Fam27 | T6SSiii_tssH | T6SSiii | VFG002480(gi:53722524) | 1 | 1 | 1 | 1 | 1 | 1 | 1 | 1 | 1 | 1 | 1 | 1 | 1 | 1 | 1 | 1 | 1 | 1 | 1 |
| Fam1 | T6SSiii_tssI | T6SSiii |  | 1 | 1 | 1 | 1 | 1 | 1 | 1 | 1 | 1 | 1 | 1 | 1 | 1 | 1 | 1 | 1 | 1 | 0 | 0 |
| Fam28 | T6SSiii_tssI | T6SSiii |  | 2 | 2 | 0 | 0 | 1 | 1 | 1 | 1 | 3 | 2 | 4 | 3 | 3 | 1 | 1 | 1 | 1 | 1 | 1 |
| Fam24 | T6SSiii_tssK | T6SSiii |  | 1 | 1 | 1 | 1 | 1 | 1 | 1 | 1 | 1 | 1 | 1 | 1 | 1 | 1 | 1 | 1 | 1 | 1 | 1 |
| Fam23 | T6SSiii_tssN | T6SSiii |  | 1 | 1 | 1 | 1 | 1 | 1 | 1 | 1 | 1 | 1 | 1 | 1 | 1 | 1 | 1 | 1 | 1 | 1 | 1 |
| Fam18 | T6SSiii_tssP | T6SSiii | VFG002276(gi:18309155) | 1 | 1 | 1 | 1 | 1 | 1 | 1 | 1 | 1 | 1 | 1 | 1 | 1 | 1 | 1 | 1 | 1 | 1 | 1 |
| Fam19 | T6SSiii_tssQ | T6SSiii |  | 1 | 1 | 1 | 1 | 1 | 1 | 1 | 1 | 1 | 1 | 1 | 1 | 1 | 1 | 1 | 1 | 1 | 1 | 1 |
|  |  |  |  |  |  |  |  |  |  |  |  |  |  |  |  |  |  |  |  |  |  |  |
|  |  |  |  |  |  |  |  |  |  |  |  |  |  |  |  |  |  |  |  |  |  |  |
|  |  |  |  |  |  |  |  |  |  |  |  |  |  |  |  |  |  |  |  |  |  |  |

**TABLE S6**. Protein name, Pfam family name, Pfam number and description of all protein profiles searched in *Elizabethkingia* genomes in order to identify putative capsular polysaccharide synthesis clusters.

| **Protein Name** | **Pfam Name** | **Pfam Number** | **Description** |  |
| --- | --- | --- | --- | --- |
| Capsule synthesis |  |  |  |  |
| KpsT | ABC_tran | PF00005 | ABC transporter |  |
| KpsM | ABC2_membrane | PF01061 | ABC-2 type transporter | |
|  |  |  |  |  |
| Wzy | Wzy_C | PF04932 | Polymerase |  |
| Wzy | O-antigen_lig | PF13425 | Polymerase |  |
| Wzx | Polysacc_synt_3 | PF13440 | Flippase |  |
| Wzx | Polysacc_synt_C | PF14667 | Flippase |  |
|  |  |  |  |  |
| Capsule export |  |  |  |  |
| Wza (OMP) | Poly_export | PF02563 | Outer membrane export protein | |
| Wzb | LMWPc | PF01451 | Low molecular weight phosphotyrosine protein phosphatase | |
| Wzc (IMP) | Wzz | PF02706 | Chain length regulator | |
| Wzc (IMP) | AAA31 | PF13614 | Translocation of macromolecules | |
|  |  |  |  |  |
| Sugar modifying-enzymes |  |  |  |  |
| Glycosyl transferase | Glycos_transf_2 | PF00535.23 | Glycosyl transferase family 2 | |
| Epimerase | Epimerase | PF01370.18 | NAD dependent epimerase/dehydratase family | |
| GDP-mannose 4,6 dehydratase | GDP_Man_Dehyd | PF16363.2 | GDP-mannose 4,6 dehydratase | |
| Acetyltransferase | Acetyltransf_1 | PF00583.22 | Acetyltransferase (GNAT) family | |
| Bacterial sugar transferase | Bac_transf | PF02397 | Bacterial sugar transferase | |
| Nucleotidyl transferase | NTP_transferase | PF00483 | Nucleotidyl transferase | |

**Supplementary figures**

**Figure S1.** Phylogenetic analysis of *Elizabethkingia* isolates based on 16S rRNA sequences. a. Phylogenetic tree with bootstrap-based support (500 replicates) >70% indicated at branches. b. Detail of the multiple 16S rRNA sequence alignment (aligned characters 963-1,033).

**Figure S2**. Gene tree of chloramphenicol acetyltransferase (CAT) genes. Blue: sequences from Schwarz et al. 2004, FEMS Microbiology Reviews 28:519–542; magenta: sequences from *Elizabethkingia* strains; black: closely related homologous sequences selected from RefSeq. NCBI accession ids are given on the right. Bootstrap-based supports (500 replicates) >70% are indicated at branches.

**Figure S3**. Distribution of CRISPR-Cas systems in *Elizabethkingia* genus*.* White indicates absence of the trait and black indicates its presence. The numbers reported on the right correspond to the number of spacers identified in each genome.

**Figure S4**. Gene-by-gene comparison of the *Elizabethkingia* genomes based on 1,546 loci included in the core genome multilocus sequence typing (cgMLST) scheme. a: UPGMA dendrogram computed from the matrix of allelic mismatches distances (i.e., the proportion of distinct alleles among each pair of profiles). b: Minimum spanning tree of the Singapore lineage. The number of allelic mismatches between each displayed link is given. c: Minimum spanning tree of the African lineage, which differ by 4 alleles out of 1,546.
